# Commutability study on three CRMs evaluating their suitability for calibration, trueness verification and statistical quality control of methods measuring metal concentrations in human blood

**DOI:** 10.1007/s00216-025-05751-0

**Published:** 2025-02-04

**Authors:** Thomas P. J. Linsinger, Guy Auclair, Liesbet Deprez

**Affiliations:** Joint Research Centre, European Commission, Retieseweg 111, 2440 Geel, Belgium

**Keywords:** Commutability, Certified reference material, Metrology

## Abstract

The commutability of three certified reference materials for Cd, Cr, Hg, Ni, Pb and Tl in blood was evaluated for the combination of digestion ICP-MS with dilution ICP-MS or dilution GFAAS using the difference in bias approach. Maximum non-commutability bias (MANCB) was derived from the median of the tolerance limits set by six proficiency testing (PT) providers. The evaluation showed that this criteria are too strict and that either unfeasible high number of measurements or a larger MANCB is required to demonstrate commutability. The effect of increasing the MANCB on the maximum tolerable measurement uncertainty was found small to moderate demonstrating the commutability of the materials when used as a calibrator. Also the inclusion of the uncertainty of non-commutability into the measurement uncertainty resulted in only small increases of the measurement uncertainty, demonstrating the suitability of the materials for trueness control and method validation. The bias of the three CRMs was for most elements within the 99% confidence limits of the bias of the patient samples, demonstrating the suitability of the materials for statistical quality control.

**Graphical abstract:**

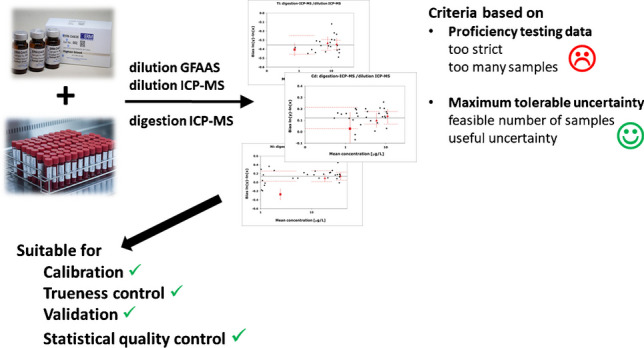

## Introduction

Commutability of a reference material (RM) is defined as “property of a reference material, demonstrated by the closeness of agreement between the relation among the measurement results for a stated quantity in this material, obtained according to two given measurement procedures, and the relation obtained among the measurement results for other specified materials” [1]. In simpler words, it means that the same amount of measurand in the reference material and in routine samples produces the same measurement response in different measuring systems. In this context, it is important to note that commutability is not a universal property, but a property when comparing two measurement procedures. An RM is commutable for one or several pairs of measurement procedures. This is especially relevant for RMs with a certified value obtained by one measurement procedure but their main use is calibration or trueness verification of different measurement procedures. This is often the case in the field of clinical chemistry, where highly accurate but time-consuming reference measurement procedures exist. The amount of time needed for executing these measurement procedures precludes the use of these measurement procedures for the high sample throughput in clinical settings. Therefore, different measurement procedures are usually applied in daily routine which may target different analytes than the reference measurement procedures. For example, a routine immunoassay targets a part of the complex 3D structure of the protein (epitope) whereas the reference measurement procedure quantifies the protein concentration by measuring a signature peptide (obtained after tryptic digestion of the protein) with mass spectrophotometry. Due to this difference in target analyte, the response between the RM and routine samples may vary.

The effect of non-commutability can be stark: whereas a reference material aims to harmonise measurement results and to improve comparability, using a non-commutable RM for calibration can introduce an additional bias and therefore worsen the comparability of results; similarly, a non-commutable RM will not be suitable for trueness control [2]. For this reason, current standards for general RM production require the assessment of commutability, where appropriate [3], and the ISO standard for certified reference materials (CRMs) for in vitro medical devices explicitly requires the evaluation of commutability for materials used as a calibrator or trueness control [2].

While the concept of commutability is best developed and most-widely used for clinical CRMs, it is definitely not limited to the clinical field. For example, a measurement procedure based on thermogravimetry for the determination of volatility of coal gave a different result than the ISO standard method for a CRM of hard coal [4]. The reason is that the thermogravimetry method had been calibrated with other types of hard coals and the CRM was therefore for this method not representative of the coals the laboratory normally measured, meaning that this hard coal CRM was not commutable for the combination of the ISO standard method and the in-house thermogravimetry method.

Assessment of commutability involves measurement of a number of real samples together with several units of one or more CRMs using at least two measurement procedures. Several approaches have been developed to demonstrate the commutability of CRMs when used as a calibrator. CLSI-EP30 version 1 [5] suggests performing a regression analysis between the results of the two measurement procedures for the clinical samples and checking whether the results for the CRMs lie within the 95% prediction interval of this regression line. One disadvantage of this approach is that the width of the prediction interval is determined by the scatter on the clinical samples and not by the analytical requirements. If that scatter is large, basically any CRM will be commutable. A second disadvantage is that there is no estimation of the uncertainty associated with the commutability assessment. An updated version of the guideline was recently released [6] that aims to address these issues. The International Federation of Clinical Chemistry and Laboratory Medicine (IFCC) proposed an assessment using the difference in bias between the CRMs and patient samples [7, 8]. In this approach, the bias between the two procedures is evaluated for the clinical samples and the CRMs. A maximum non-commutability bias (MANCB) is defined. A CRM is deemed commutable if the bias and its confidence interval are completely inside the interval ± MANCB and are non-commutable if they are completely outside the interval ± MANCB. In cases where the bias and its confidence interval overlap the interval ± MANCB, no clear assessment is possible and the result is “inconclusive”. A critical part of the commutability assessment is the choice of the MANCB. Miller and co-workers [9] recommend choosing a MANCB that does not significantly increase the measurement uncertainty, namely 3/8 of the tolerated standard measurement uncertainty. The reasoning behind this limit is that at this uncertainty, the uncertainty of the commutability assessment does not significantly contribute to the overall measurement uncertainty. The Guide to the expression of uncertainty in measurement [10] states that uncertainty contributions less than 1/3 of the largest contribution of a combined uncertainty are negligible, which is largely in line with the fraction of 3/8 given above.

Unlike calibrators which are produced as large batches at a given concentration, quality control materials of different rounds of proficiency tests (PT) have different concentrations in each round. This means an aggregate commutability assessment for many rounds is necessary. While it is reasonable that different batches prepared in the same way are equally commutable, this needs to be checked. A second complication is that laboratories applying all possible measurement procedures can participate in a PT, which goes against the idea of assessing commutability method combination by method combination. Sandberg and colleagues therefore proposed basing the commutability assessment of the statistical distribution of results of clinical samples of two methods and propose that a material for quality assurance is regarded as commutable if it falls within the 99% prediction interval of the patient samples [11].

Three whole blood CRMs named ERM-DA634, ERM-DA635 and ERM-DA636 were recently released with certified values for Cd, Cr, Hg, Ni, Pb and Tl [12]. It may seem that CRMs certified for element mass concentrations are unlikely to suffer from non-commutability. Routine samples for elements are usually non-frozen whole blood samples with physiological Ca concentrations. In contrast, there are several processing steps essential to guarantee the homogeneity and stability of the CRM that might lead to different responses between the CRMs and routine samples: (i) the blood cells are lysed and cell membranes and other debris are removed; (ii) during the defibrination step, relatively high Ca concentrations are added to buffer the ethylenediaminetetratacetic acid (EDTA) added as anticoagulant; (iii) the relevant metals were spiked to obtain the high concentrations; (iv) the material was freeze-dried. Most measurement procedures in the characterisation study of the CRMs involved a digestion step, whereas routine measurement procedures for elements in blood usually employ a dilute-and-measure approach: the whole blood is diluted and then directly measured by inductively coupled plasma mass spectrometry (ICP-MS) or graphite furnace atomic absorption spectrometry (GFAAS). The study to assign the certified values (“characterisation study”) was conducted as an intercomparison between laboratories of demonstrated competence. It included one dataset each of these dilution approaches and no significant difference between their results and the results from digestion methods were observed. However, no comparison with routine samples was performed in the characterisation study. Therefore, a dedicated commutability study was conducted.

The foremost aim of the study was of course assessment of the commutability of the three CRMs to evaluate whether they are suitable for calibration trueness control and statistical quality control. In addition, the suitability of the approaches described in the literature for assessing the commutability of CRMs for trace metals was to be evaluated.

## Materials and methods

### Materials for the commutability study

#### Certified reference materials

Frozen human EDTA blood was obtained from In.vent Diagnostica (Henningsdorf, DE). The samples were obtained from donors without specific risk profile for elevated levels of metal in blood. All donations were collected according to the principles of the Declaration of Helsinki and Taipei, processed and verified in a way that is ethically and legally compliant for the purposes of diagnostic research and development, production and quality assurance. To each donation, 2.21 mmol CaCl_2_ (99.99% on metal basis; ThermoScientific, Kandel, Germany) was added to each donation to bind the EDTA used as anticoagulant. Each donation was then lysed individually using an ultrasonic probe and left for 2–3 h at 37 °C for clotting. The lysates were pooled, centrifuged at 1200 G (Sorvall RC6 + , ThermoFisher Scientific, Waltham, USA) and filtered using a 0.45-µm polyethersulfone filter (ThermoFisher Scientific, Waltham, USA) followed by a second filtration through a 0.22-µm polyethersulfone filter (VWR, Haasrode, BE).

Three different materials at three concentration levels were prepared, with the CRM codes ERM-DA634 (low level), ERM-DA635 (medium level) and ERM-DA636 (high level), respectively. Samples were spiked with a solution of metals in slightly acidified water to obtain three different concentration levels dispensed into 5-mL amber glass vials and the content was lyophilised. After freeze-drying, the vacuum was broken with Ar and the vials were closed with silicone stoppers and capped.

The materials were tested for homogeneity and stability and certified values were assigned based on an interlaboratory comparison of laboratories of demonstrated competence. Most laboratories in this intercomparison used acid digestion. The preparation of the materials, including the homogeneity and stability assessment and the characterisation study are described in detail in [12] and the certified values for the three materials are shown in Table 1.

**Table 1:** Certified mass concentrations in µg/L of the reference materials ERM-DA634, ERM-DA635 and ERM-DA636

	ERM-DA634	ERM-DA635	ERM-DA636
Cd	1.29 ± 0.09	5.7 ± 0.4	10.9 ± 0.6
Cr	1.5 ± 0.4	22.0 ± 2.4	42 ± 4
Hg	1.6 ± 0.3	25.5 ± 2.9	56 ± 6
Ni	Not certified	18.3 ± 1.6	36 ± 5
Pb	18.6 ± 1.7	182 ± 11	0.44 × 10^3^ ± 0.04 × 10^3^
Tl	0.81 ± 0.08	8.3 ± 0.7	16.6 ± 1.4

#### Clinical samples

Twenty individual donations of donors with a higher risk of elevated blood element levels were obtained from In.Vent Diagnostica (GmbH, Hennigsdorf, DE) and three aliquots of 10 mL each were taken of each donation. The risk factors were smoking, amalgam fillings and working in metal processing industries. In addition, 22 aliquots of approximately 30 mL blood were spiked at 22 different element levels to ensure to cover the complete concentration range. Each spiked sample was mixed and split into three aliquots of 10 mL each. Timing of the preparation was crucial. The donations were taken on day 1 and sent to the JRC Geel on the same day and arrived in the late afternoon. The samples were spiked on day 2 in the morning and were sent on the same afternoon to the laboratories performing the measurements. All shipments were performed cooled to ensure the integrity of the samples.

### Setup of the commutability study

The total study consisted of 42 clinical samples and six samples of each of the three CRMs, 18 CRM samples in total. A randomised sequence was generated that interspersed the CRM samples with the clinical samples. Laboratories were instructed to perform three replicate measurements of each sample in the specified sequence. The three replicates were performed immediately one after the other from the same sample preparation.

### Measurement procedures

#### Digestion ICP-MS

Measurements were performed by ALS Scandinavia (Umeå, SE). 500 mg of sample was taken and HNO_3_ was added. The samples were digested in a closed microwave digestion system and quantified on a sectorfield ICP-MS using calibration curves obtained from standard solutions in acid.

#### Dilution ICP-MS

Measurements were performed by the Medizinische Laboratorien Düsseldorf (Düsseldorf, DE). Aliquots of 200 µL were diluted with 3800 µL of a basic diluent consisting of iso-propanol, ammonia, Triton-X and ETDA. The resulting solution is directly analysed by ICP-MS (NexION, PerkinElmer) using calibration curves obtained from standard solutions in acid.

#### Dilution GFAAS

The measurements were performed by the Medizinisches Labor Ostsachsen MVZ GbR (Görlitz, DE). Aliquots of 400 µL blood were mixed with 700 µL HNO_3_ and 1 mL 0.1% Triton-X-100. The sample was centrifuged and the supernatant was quantified using a GFAAS (PinAAcle 900 T, PerkinElmer) using matrix-matched standards. The method was used only for the Cd and Pb concentrations.

### Evaluation of the commutability study

#### Study setup

The commutability study was evaluated using the difference in bias approach described by the IFCC working group on commutability [8] using the example file for the commutability documentation from the supplementary material of that publication. The evaluation was performed separately for each element and for the combinations digestion ICP-MS/dilution ICP-MS and digestion ICP-MS/dilution GFAAS. Commutability of the dilution method with the digestion method was of key interest, as the certified values were largely based on digestion methods where routinely dilution methods are applied. The procedure is shortly described below.

In the first step, all samples that gave a result below the reporting limit in any of the methods compared for this element were removed. Then, all obviously outlying results of clinical samples were removed, before the decision was taken whether the results should be log-transformed. The distribution of the (transformed) biases was checked using normal probability plots for obvious deviations of normality, as analysis of variance (ANOVA) requires roughly normally distributed data. ANOVAs were performed to identify between-sample differences, and finally, the commutability was assessed.

The absolute value of the bias and its expanded uncertainty were calculated using the coverage factor of 1.9 as suggested in [8] for all elements and materials.

#### Commutability when used as a quality control material

It was checked whether the mean bias for each element and RM was within the interval ${b}_{CS}\pm {t}_{1\;\%,\;{n}_{cs}-1}\bullet \frac{{s}_{bcs}}{\sqrt{{n}_{RM}}}$ with *b*_cs_ being the mean bias of the clinical samples, *s*_bcs_ the standard deviation of the bias of the clinical samples, n_cs_ and n_RM_ the number of the clinical samples and reference material, respectively, and t_1%, ncs-1_ the t-factor for a 99% confidence level. s_bcs_ is divided by the square root of the number of reference material samples as the comparison is performed on the mean bias of n_RM_ samples.

#### Commutability when used as calibrators

To our knowledge, no widely accepted regulatory limits for the maximum tolerable measurement uncertainty exist. On the other hand, medical testing is strictly regulated and participation in proficiency tests (PT) is often required. Therefore, the maximum acceptable deviation in PTs was used as proxy for the maximum tolerable measurement uncertainty. A number of European Union, UK, American and Australian PT providers were contacted for their performance limits for the respective elements in blood which are given in Table 2.

**Table 2:** Maximum deviation limits used by various PT providers

	Instand, Germany [13]	RCPAQAP, Australia [14]^a^	CLIA, USA [15]	G-EQUAS, Germany^b^ [16]	New York State, USA [17]	UK-NEQUAS, UK [18]	OELM, Austria, Belgium, France, Italy, Spain, The Netherlands [19]
Cd	36%	0.34 µg/L (< 2.2 µg/L) 15% (> µg/L)	-	All: 15%	max (1 µg/L, 15%)	0.34 µg/L (< 3.4 µg/L) 10% (> 3.4 µg/L)	max (0.32 µg/L, 15%)
Cr	36%	0.15 µg/L (< 1.3 µg/L) 15% (> 1.3 µg/L)	-	All: 15%	max (2 µg/L, 20%)	2.1 µg/L (< 10 µg/L) 20% (> µg/L)	max (0.32 µg/L, 15%)
Hg	36%	1.8 µg/L if c < 12 µg/L 15% (> 12 µg/L)	-	ERM-DA634: 30% ERM-DA635, ERM-DA636: 20%	max (2 µg/L, 20%)	2 µg/L (< 13 µg/L) 15% (> 13 µg/L)	max (0.61 µg/L, 20%)
Ni	36%	-	-	ERM-DA634: 30% ERM-DA635, ERM-DA636: 15%	-	-	-
Pb	36%	10.5 µg/L (< 100 µg/L) 10% (> 100 µg/L)	max (20 µg/L, 10%)	ERM-DA634: 20% ERM-DA635, ERM-DA636: 15%	Max (20 µg/L, 10%)	30 µg/L (< 100 µg/L) 10% (> 100 µg/L)	max (20 µg/L, 10%)
Tl	-	0.41 µg/L (< 2 µg/L) 10% (> 2 µg/L)	-	ERM-DA634: 30% ERM-DA635: 20% ERM-DA636: 15%	-	0.005 µg/L (< 0.04 µg/L) 25% (> 0.04 µg/L)	max (0.17 µg/L, 13%)

^a^Concentration limits for RCPAQAP were converted from nmol/L to µg/L

^b^The German EQUAS scheme does not operate fixed tolerance levels but sets levels for each PT separately. The reference data for PTs 2012–2021 were downloaded from the website https://app.g-equas.de/web/, plotted against the concentrations and appropriate levels were set according the certified concentrations in ERM-DA634, ERM-DA635 and ERM-DA636

Results in the PTs listed in Table 2 are considered questionable if they exceed these maximum deviations. In turn, this means that the maximum acceptable expanded uncertainty corresponds to these limits. This means that the MANCB as defined in [9] is 3/8 of these limits. The median of these values was chosen as MANCB, leading to the values given in Table 3.

**Table 3:** MANCB in µg/L for the commutability assessment of the three reference materials ERM-DA634, ERM-DA635 and ERM-DA636

	ERM-DA634	ERM-DA635	ERM-DA636
Cd	0.13	0.33	0.63
Cr	0.27	1.51	2.82
Hg	0.45	1.81	3.67
Ni	0.30	2.46	3.53
Pb	7.50	7.50	15.18
Tl	0.079	0.47	0.80

### Estimation of the number of CRM and clinical samples needed to reach commutability

The standard uncertainty of the bias of the CRM *u*(B_RM_) was taken from the evaluation of the commutability study and was calculated as

$${u(B}_{RM})=\sqrt{\frac{{s}_{mean x}^{2}+{s}_{mean y}^{2}}{p}}$$

with *s*_mean x_ and *s*_mean y_ the standard deviations of the CRM sample means for the two methods x and y, respectively, and *p* the number of CRM samples. The standard uncertainty of the bias of the clinical samples *u(B*_*CS*_*)* was calculated as

$$u\left({B}_{CS}\right)=\sqrt{\frac{{s}_{{B}_{CS}}^{2}}{q}}$$

with *s*_*BCS*_ the standard deviation of the bias of the clinical samples and q the number of the clinical samples.

The uncertainty of the difference of the biases from the CRMs to the clinical samples *u(D*_*RM*_*)* was calculated as.

$$u\left({D}_{RM}\right)=\sqrt{u({B}_{RM}{)}^{2}+u({B}_{CS}{)}^{2}}$$

Assuming an ideally commutable material with exactly the same bias as the clinical samples, the observed difference in biases is expected to have an average of 0 and a standard deviation of *u(D*_*RM*_*)*. For the assessment of the commutability, the absolute value of the difference in bias is added to the expanded uncertainty of the bias. The expected value of the absolute value of a variable with an average of 0 and a standard deviation s is s $*\sqrt{\frac{2}{\pi }}$ [20]. The expected value of the bias was therefore calculated as $u\left({D}_{RM}\right)*\sqrt{\frac{2}{\pi }}$.

The minimum value of samples needed to be able to demonstrate commutability was calculated using the solver function in Microsoft Excel by minimising the sum of *p* and *q* with the constraints that *p* and *q* are integers, *p* ≥ 1, *q* ≥ 30 as recommended in [8] and that *U(D*_*RM*_*)* + $u\left({B}_{RM}\right)*\sqrt{\frac{2}{\pi }}$  < MANCB.

### Effect increasing the MANCB to the achievable uncertainty

For each element and material, the MANCB to achieve the assessment “commutability” was calculated and converted into a percentage of the average value. According to [9], the uncertainty attributable to non-commutability (*u*_nc_) is MANCB/√3. The *u*_nc_ was calculated for all elements and materials as was the maximum relative standard uncertainty *u*_max_ corresponding to the MANCB in Table 3. The new expanded uncertainty U was calculated as $U=2*\sqrt{{u}_{max}^{2}+{u}_{nc}^{2}}$.

### Increase of the uncertainty when the materials is used for trueness control

An aggregate expanded measurement uncertainty for each element and each CRM was obtained from the results of the characterisation study. The aggregate uncertainty *u*_agg_ was estimated as ${u}_{agg}=\sqrt{\frac{\sum {u}_{i}^{2}}{p}}$, with *u*_i_ being the expanded uncertainty of the p-individual datasets obtained for the respective element and CRM.

ISO 21748 [21] describes the repeatability and reproducibility information can be used to estimate measurement uncertainties. Measurement uncertainties were estimated for each element and CRM using repeatability standard deviation (*s*_r_) and the between-laboratory standard deviation (*s*_ip_) from the characterisation intercomparison [12]. It was checked if the individual *u*_agg_ for each element and CRM were credible by comparing them to the uncertainties estimated from the intercomparison. Estimates of *u*_agg_ smaller than the uncertainties estimated from the intercomparison indicate a tendency of the participating laboratories to underestimate the measurement uncertainties.

The *u*_nc_ was then added to *u*_agg_ to see how much potential non-commutability would increase the measurement uncertainty.

## Results and discussion

Log transformation of the data resulted in precision that was constant over the concentration range. The normal probability plots did not show any obvious deviations from normality.

**Fig. 1:**
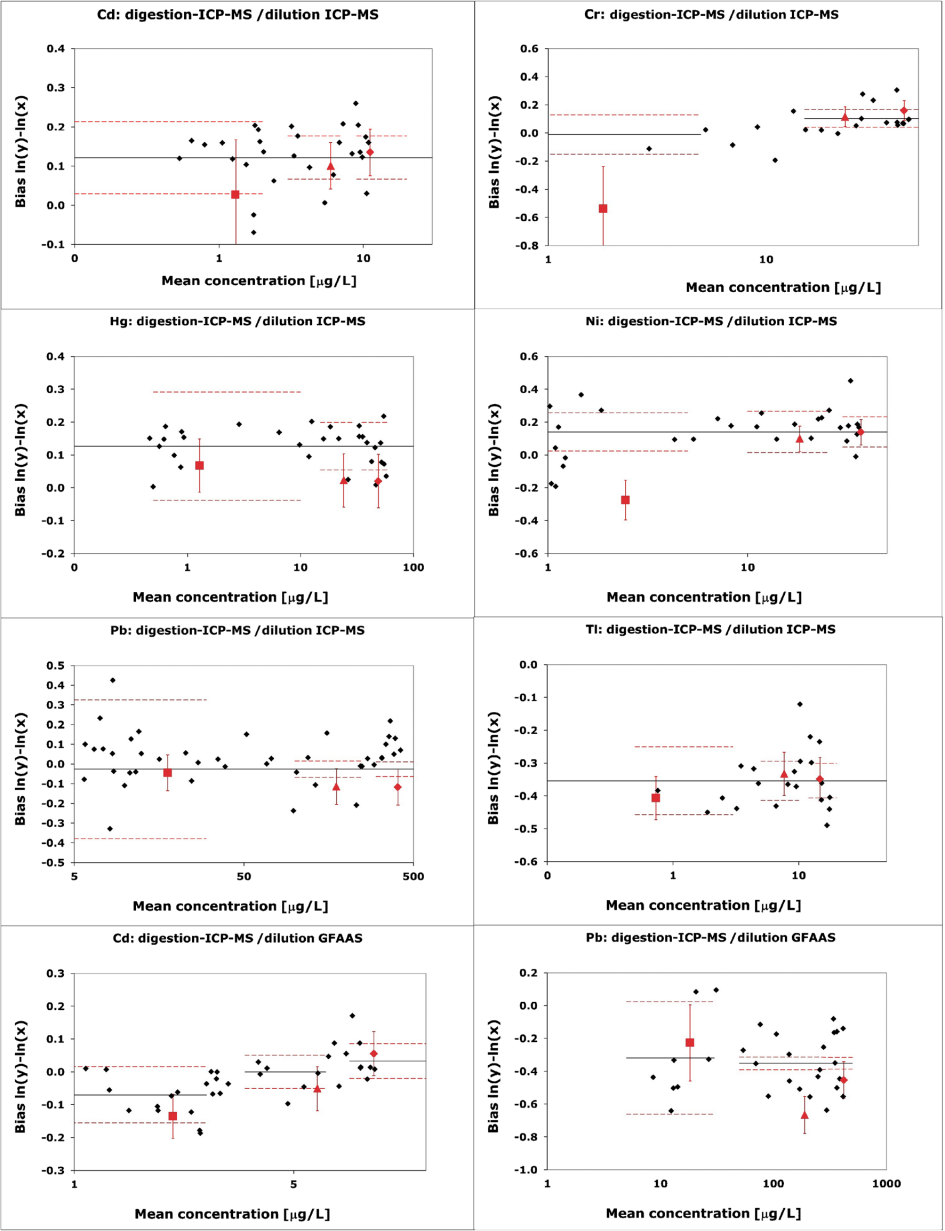
Commutability graphs for the combination dilution ICP-MS (*x*)/digestion ICP-MS (*y*) and dilution GFAAS (x)/digestion ICP-MS (y). Blue: Results of the clinical samples. Red squares: ERM-DA634. Red triangles: ERM-DA635. Red diamonds: ERM-DA636. Note that commutability for Cr for ERM-DA634 cannot be evaluated (no clinical samples in the same concentration range). The same is true for ERM-DA634 for the Ni mass concentration, which is not certified

The results of the statistical evaluations for the suitability as calibrator are given in Table 4; the graphs showing the results of the CRMs, the clinical samples and the MANCB are given in Fig. 1. Of the 40 comparisons, 35 showed no significant non-commutability bias meaning that the 95% confidence interval of the bias of the CRM overlapped with bias of the clinical samples.

**Table 4:** Results of the statistical evaluations of the commutability of ERM-DA634, ERM-DA635 and ERM-DA636 for dilution ICP-MS and dilution GFAAS versus digestion-ICP-MS

Element	Method comparison^a^	Outcome commutability assessment		
		ERM-DA634	ERM-DA635	ERM-DA636
Cd	Dilution ICP-MS	Inconclusive	Inconclusive	Inconclusive
	Dilution GFAAS	Inconclusive	Inconclusive	Inconclusive
Cr	Dilution ICP-MS	Concentration in the CRM below the lowest clinical samples: no evaluation possible	Inconclusive	Inconclusive
Hg	Dilution ICP-MS	Commutable	Inconclusive Significant non-commutability bias	Inconclusive Significant non-commutability bias
Ni	Dilution ICP-MS	Not certified, evaluation not meaningful	Commutable	Commutable
Pb	Dilution ICP-MS	Commutable	Inconclusive	Inconclusive
	Dilution GFAAS	Commutable	Not commutable Significant non-commutability bias	Inconclusive
Tl	Dilution ICP-MS	Inconclusive	Inconclusive	Inconclusive

^a^All comparisons are performed against digestion ICP-MS

For most materials, there are significant between-sample effects. This is to be expected as an additional error component is introduced by the sample preparation. Small differences in the amount of sample taken for dilution or small variations in the digestion introduce between-sample variations that show as position effects. These position effects also mean that the three replicates per sample preparation are not independent and that there are therefore indeed only three independent results per CRM.

For the combination dilution ICP-MS and digestion ICP-MS, ERM-DA634 is deemed not commutable for Ni. This may be an effect of the laboratory performing the digestion ICP-MS and was already noticed during the characterisation exercise: the laboratory uses a sector field ICP-MS and a different isotope than the other laboratories using ICP-MS (62 rather than 60). Because dataset of this laboratory was also significantly lower than the data from the other laboratories, the Ni mass concentration was not certified in ERM-DA634. The non-commutability is therefore not relevant.

**Fig. 2:**
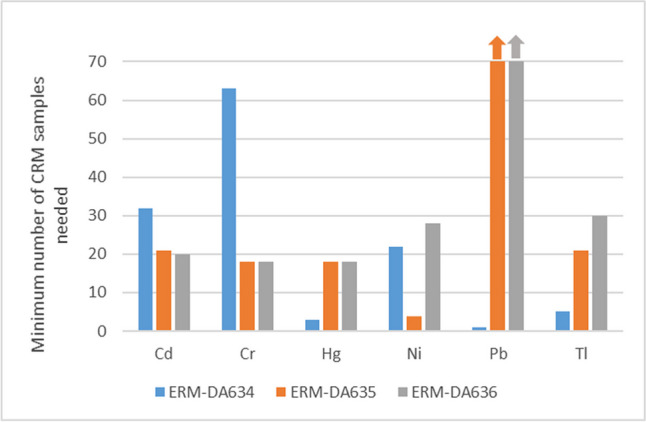
Minimum number of CRM samples required to be able to demonstrate commutability. The values for Pb are 81,946 and 135 for ERM-DA635 and ERM-DA636, respectively

The assessment for most of the other elements is inconclusive. This is largely due to the very low MANCB compared to the uncertainty of the commutability assessment. The uncertainty of the bias of the clinical samples is, with the exception of Ni, always smaller than the uncertainty of the reference materials. Reducing the uncertainty of the commutability assessment by changing the number of samples is possible. The optimisation showed that the number of clinical samples would not have to be increased significantly: the optimal number of clinical samples was for all elements except Pb between 30 and 36. However, as shown in Fig. 2, for most elements, the number of CRM samples would have to be increased from six to about 20 to be able to demonstrate commutability. This is a significant burden, especially if, like in this case, several CRMs are tested. Commutability for Pb for ERM-DA635 and ERM-DA636 is an extreme case: The optimisation resulted in an optimal number of 78 (ERM-DA635) and 164 (ERM-DA636) clinical samples Worse still, 135 CRM samples of ERM-DA636 and 81,942 samples of ERM-DA635 would be needed to be able to demonstrate commutability, which is clearly not feasible These high numbers show that that a MANCB of 3/8 of the tolerated deviations in PTs is certainly not realistic for Pb and also difficult to apply for the other elements.

**Fig. 3:**
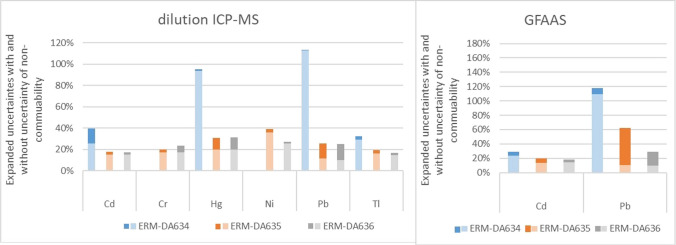
Maximum tolerable uncertainty without (light bars) and with (darker bars) inclusion of the uncertainty of non-commutability

When the initially suggested MANCB turns out to be unrealistic, Miller and colleagues suggested using a larger value for MANCB and investigating the effect of this larger value on the maximum tolerated uncertainty [9]. Figure 3 shows how the maximum uncertainty would change if the uncertainty of non-commutability was included. Inclusion of the uncertainty of non-commutability results of course in an increase of the expanded uncertainties, but even with this additional uncertainty contribution, the uncertainties remain sufficiently small to be fit for purpose. The small to moderate effect of a larger MANC on the maximum tolerated uncertainty shows that the materials are suitable as calibrators.

**Fig. 4:**
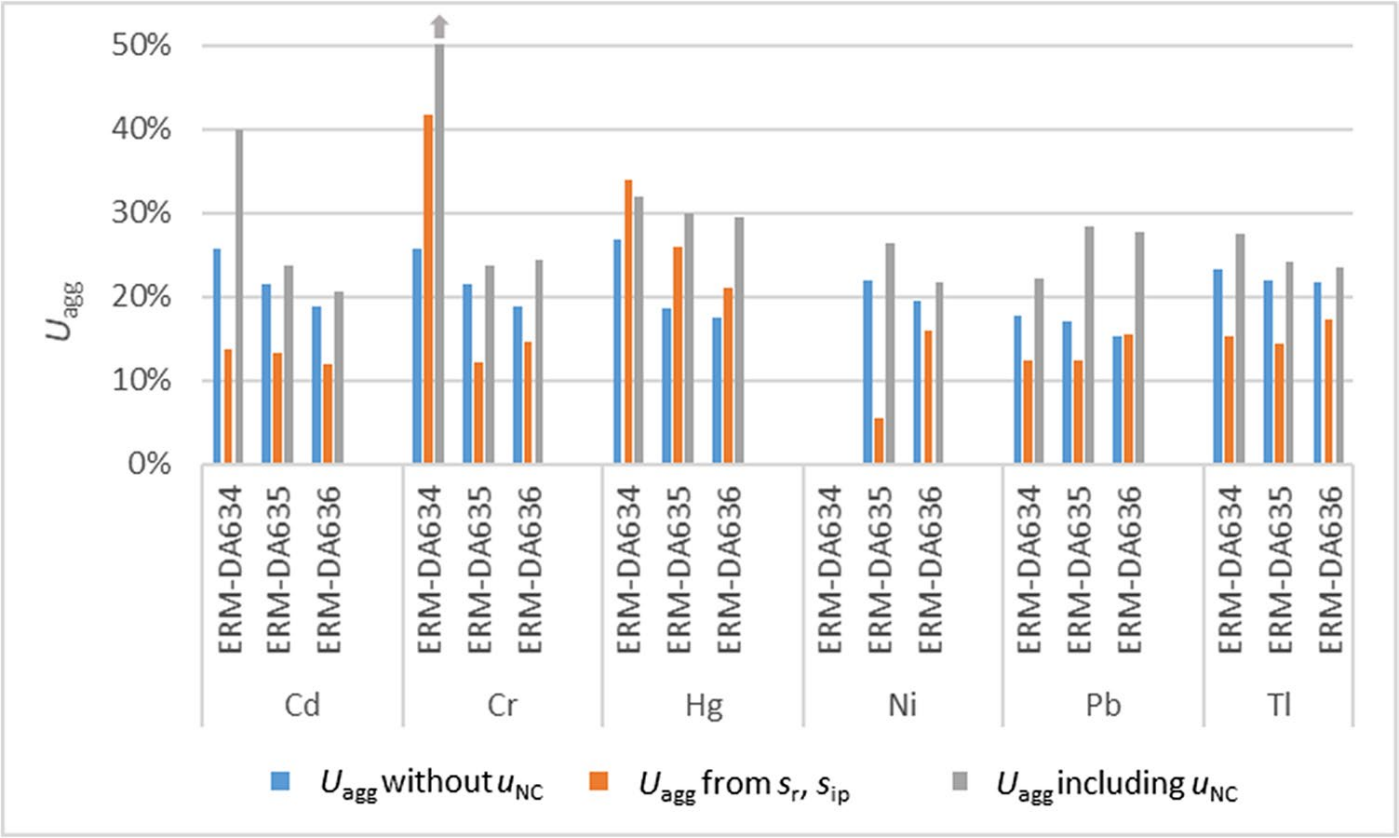
Expanded measurement uncertainties *U*_*agg*_ (aggregate uncertainty) with or without including *u*_*nc*_. s_r_, s_ip_: repeatability and between-laboratory standard deviation. All uncertainties are expanded uncertainties with *k* = 2. The uncertainty for Cr for ERM-DA634 is 150%

Figure 4 shows the expanded uncertainties calculated from the uncertainties as stated by the laboratories in the characterisation exercise of CRM production (*u*_agg_), estimated using the within- and between-laboratory standard deviation (from *s*_r_, *s*_ip_) from the characterisation exercise and *u*_agg_ including the non-commutability uncertainty. In most cases, the uncertainties estimated from the within- and between-laboratory standard deviation were smaller than the aggregate uncertainty calculated from the uncertainties estimated by the laboratories. This shows that the laboratories included more than the pure variation between laboratories in their estimate and confirms the reliability of the laboratories’ estimates. Adding the *u*_NC_ as an additional uncertainty contribution to *u*_agg_ also increases the uncertainties but these increases of uncertainties are in most cases small and smaller than the differences between the two approaches for estimating measurement uncertainties. The exception again is Pb for ERM-DA635 and ERM-DA636. Even after adding the non-commutability uncertainty for Pb to the uncertainties for ERM-DA635 and ERM-DA636, the expanded uncertainties would still be sufficiently small to be fit for purpose.

As the effect of inclusion of *u*_NC_ into the measurement uncertainty is small, the materials are suitable for trueness control and method validation.

**Fig. 5:**
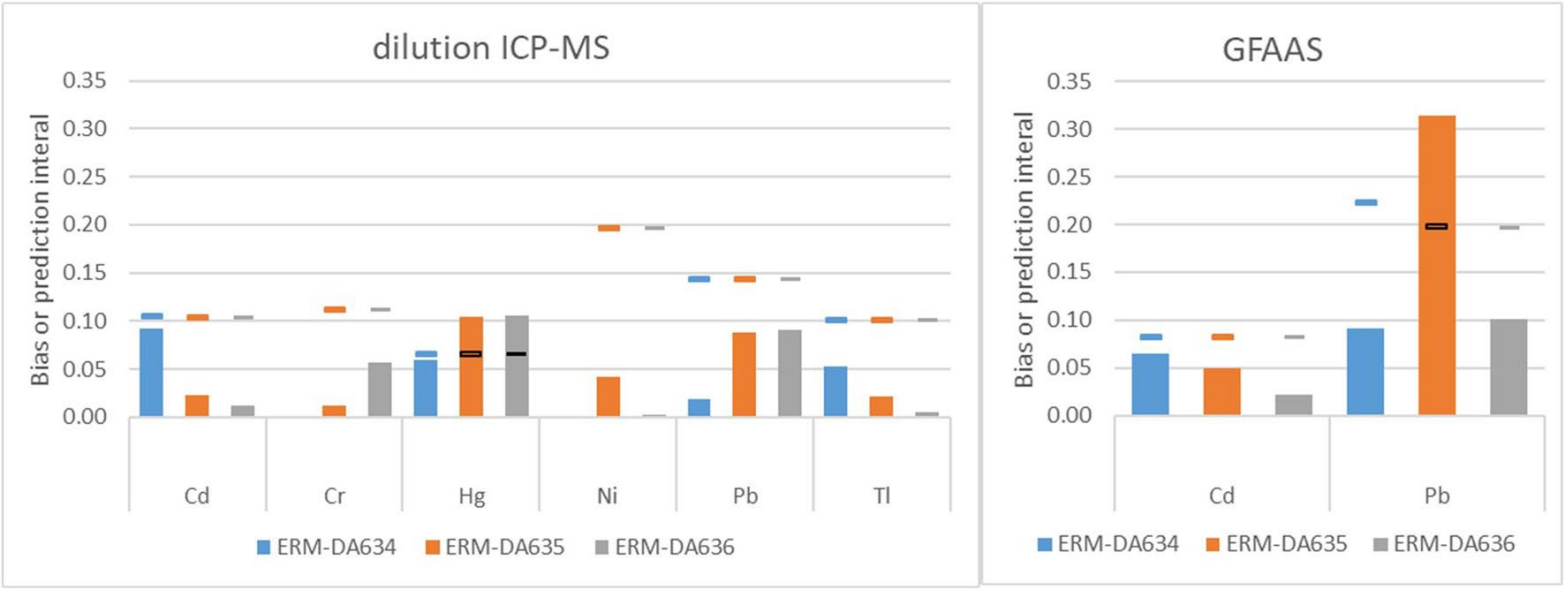
Comparison of the bias (bars) with the prediction intervals of clinical samples (lines)

As shown in Fig. 5, the bias of all materials and elements was within the 99% prediction interval of the clinical samples for all elements except Hg (ERM-DA635, ERM-DA636) and Pb (dilution GFAAS; ERM-DA635), showing that the materials are suitable as quality control materials.

## Conclusion

The evaluation demonstrated the difficulties applying the procedures for commutability testing for measurands that do not have natural physiological limits and where the commutability must be evaluated against the measurement uncertainty. As the uncertainty of bias includes the variation of both the clinical and the CRM samples, a very high number of measurements may be required to demonstrate commutability. An increase of the maximum tolerated uncertainties may be the only possibility to obtain workable limits for the commutability assessment.

For methods with a significant sample preparation step, completely independent measurements should be performed rather than quantifying the prepared sample several times, as this will reduce the confidence interval of the bias.

After an increase of the MANCB, ERM-DA634, ERM-DA635 and ERM-DA636 were found to be commutable for dilution-ICP-MS and dilution GFAAS for Cd, Hg, Pb and Tl. ERM-DA635 and ERM-DA636 were also found commutable for dilution-ICP-MS and dilution GFAAS for Cr and Ni. All three materials are suitable for trueness control and method validation as well as for statistical quality control.

## Declarations

### Plagiarism

No data, text, or theories by others are presented as if they were the author’s own.
